# 4,4′-Dianilino-3,3′-(4-chloro­benzyl­idene)bis­[furan-2(5*H*)-one]

**DOI:** 10.1107/S1600536809021084

**Published:** 2009-06-06

**Authors:** Feng Shi, Dian-Xiang Zhou, Shu-Jiang Tu

**Affiliations:** a School of Chemistry and Chemical Engineering, Xuzhou Normal University, Xuzhou 221116, People’s Republic of China

## Abstract

In the mol­ecule of the title compound, C_27_H_21_ClN_2_O_4_, the dihedral angle between the furan rings is 67.00 (3)°. The chloro­phenyl ring is oriented at dihedral angles of 76.61 (3) and 69.36 (3)° with respect to the furan rings. An intra­molecular N—H⋯O inter­action results in the formation of an eight-membered ring with a twisted conformation. In the crystal structure, inter­molecular N—H⋯O and C—H⋯O inter­actions link the mol­ecules into a three-dimensional network, forming *R*_2_^2^(16) ring motifs. Three weak C—H⋯π inter­actions are also found.

## Related literature

For the biological activity of tetronic acid derivatives and their metabolites, see: Altenbach *et al.* (2006 ▶); Foden *et al.* (1975 ▶); Ley *et al.* (1991 ▶); Roggo *et al.* (1994 ▶); Witiak *et al.* (1982 ▶). For bond-length data, see: Allen *et al.* (1987 ▶). For hydrogen-bond ring motifs, see: Bernstein *et al.* (1995 ▶).
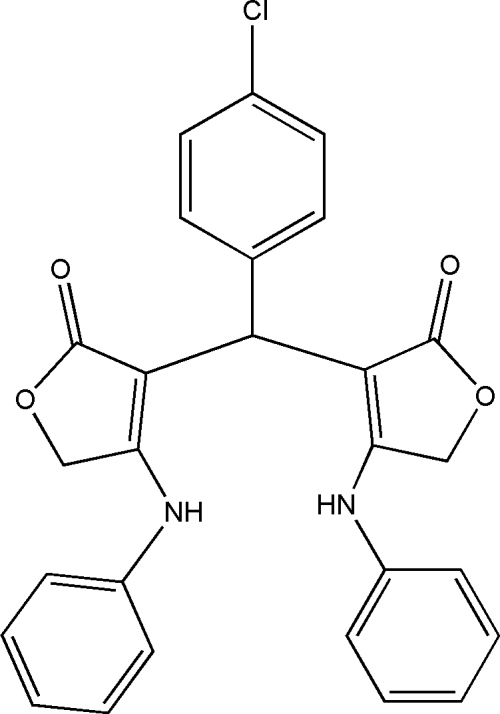


## Experimental

### 

#### Crystal data

C_27_H_21_ClN_2_O_4_
*M* *_r_* = 472.91
Monoclinic, 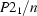

*a* = 10.973 (5) Å
*b* = 11.566 (5) Å
*c* = 17.795 (8) Å
β = 100.232 (7)°
*V* = 2222.6 (17) Å^3^
*Z* = 4
Mo *K*α radiation
μ = 0.21 mm^−1^
*T* = 298 K
0.28 × 0.15 × 0.12 mm

#### Data collection

Bruker SMART CCD area-detector diffractometer
Absorption correction: multi-scan (*SADABS*; Sheldrick, 1996 ▶) *T*_min_ = 0.943, *T*_max_ = 0.975
11368 measured reflections
3913 independent reflections
1802 reflections with *I* > 2σ(*I*)
*R*_int_ = 0.087

#### Refinement

*R*[*F* ^2^ > 2σ(*F* ^2^)] = 0.067
*wR*(*F* ^2^) = 0.251
*S* = 1.03
3913 reflections
307 parameters
H-atom parameters constrained
Δρ_max_ = 0.25 e Å^−3^
Δρ_min_ = −0.28 e Å^−3^

### 

Data collection: *SMART* (Bruker, 1998 ▶); cell refinement: *SAINT* (Bruker, 1999 ▶); data reduction: *SAINT*; program(s) used to solve structure: *SHELXS97* (Sheldrick, 2008 ▶); program(s) used to refine structure: *SHELXL97* (Sheldrick, 2008 ▶); molecular graphics: *SHELXTL* (Sheldrick, 2008 ▶); software used to prepare material for publication: *SHELXTL*.

## Supplementary Material

Crystal structure: contains datablocks global, I. DOI: 10.1107/S1600536809021084/hk2705sup1.cif

Structure factors: contains datablocks I. DOI: 10.1107/S1600536809021084/hk2705Isup2.hkl

Additional supplementary materials:  crystallographic information; 3D view; checkCIF report

## Figures and Tables

**Table 1:** Hydrogen-bond geometry (Å, °)

*D*—H⋯*A*	*D*—H	H⋯*A*	*D*⋯*A*	*D*—H⋯*A*
N1—H1⋯O4	0.86	2.05	2.797 (3)	145
N2—H2⋯O2^i^	0.86	2.09	2.907 (3)	158
C4—H4*A*⋯O4^ii^	0.97	2.24	3.206 (3)	178
C8—H8*A*⋯*Cg*5^iii^	0.97	2.98	3.721 (3)	134
C8—H8*B*⋯*Cg*4^iv^	0.97	2.91	3.617 (3)	131
C19—H19⋯*Cg*3^v^	0.93	2.90	3.766 (3)	156

Symmetry codes: (i) 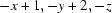; (ii) 
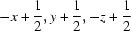
; (iii) 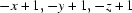; (iv) 
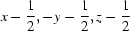
; (v) 
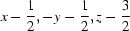
. *Cg*3, *Cg*4 and *Cg*5 are the centroids of the C10–C15, C16–C21 and C22–C27 rings, respectively.

**Fig. 1.**
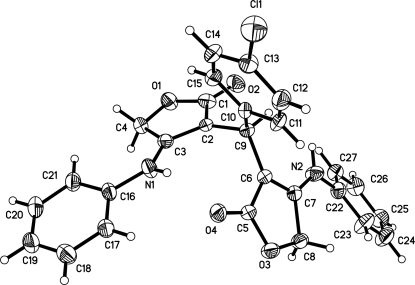
The molecular structure of the title molecule, with the atom-numbering scheme. Displacement ellipsoids are drawn at the 30% probability level.

## supplementary crystallographic information

### Comment

Tetronic acid derivatives and their metabolites have gained much attention from synthetic and medicinal chemists because of their prominent antibiotic (Ley *et al.*, 1991), antico-agulant (Witiak *et al.*, 1982), anti-inflammatory (Foden *et al.*, 1975) and anti-HIV (Roggo *et al.*, 1994) activities. Among them, open-chain derivatives of bistetronic acid, which contain two tetronic acid skeletons in one molecule, may display important pharmacological activities (Altenbach *et al.*, 2006). We report herein the crystal structure of the title compound.

In the molecule of the title compound (Fig 1), the bond lengths (Allen *et al.*,  1987) and angles are within normal ranges. Rings A (O1/C1-C4), B (O3/C5-C8), C (C10-C15), D (C16-C21) and E (C22-C27) are, of course, planar. The dihedral angles between them are A/B = 67.00 (3), A/C = 76.61 (3), B/C = 69.36 (3), A/D = 14.64 (3) and B/E = 32.61 (3) °. Intramolecular N-H···O interaction (Table 1) results in the formation of an eight-membered ring F (O4/N1/C2/C3/C5/C6/C9/H1), having twisted conformation.

In the crystal structure, intermolecular N-H···O and C-H···O interactions (Table 1) link the molecules into a three-dimensional network forming *R*_2_^2^(16) ring motifs (Bernstein *et al.*, 1995), in which they may be effective in the stabilization of the structure. There also exist three weak C—H···π interactions (Table 1).

### Experimental

The title compound was prepared by the reaction of 4-chlorobenzaldehyde (0.5 mmol) with 4-phenylamino-2,5-dihydrofuran-2-one (1 mmol) in glycol at 373 K under microwave irradiation (maximum power 200 W, initial power 100 W) for 10 min (yield; 92%, m.p. 508–509 K). Crystals suitable for X-ray analysis were obtained from an ethanol solution by slow evaporation.

### Refinement

H atoms were positioned geometrically, with N-H = 0.86 Å (for NH) and C-H = 0.93, 0.98 and 0.97 Å for aromatic, methine and methylene H, respectively, and constrained to ride on their parent atoms, with U_iso_(H) = 1.2U_eq_(C,N).

### Crystal data

**Table:**

C_27_H_21_ClN_2_O_4_	*F*(000) = 984
*M**_r_* = 472.91	*D*_x_ = 1.413 Mg m^−^^3^
Monoclinic, *P*2_1_/*n*	Melting point = 508–509 K
Hall symbol: -P 2yn	Mo *K*α radiation, λ = 0.71073 Å
*a* = 10.973 (5) Å	Cell parameters from 1079 reflections
*b* = 11.566 (5) Å	θ = 2.4–19.9°
*c* = 17.795 (8) Å	µ = 0.21 mm^−^^1^
β = 100.232 (7)°	*T* = 298 K
*V* = 2222.6 (17) Å^3^	Block, yellow
*Z* = 4	0.28 × 0.15 × 0.12 mm

### Data collection

**Table:**

Bruker SMART CCD area-detector diffractometer	3913 independent reflections
Radiation source: fine-focus sealed tube	1802 reflections with *I* > 2σ(*I*)
graphite	*R*_int_ = 0.087
φ and ω scans	θ_max_ = 25.0°, θ_min_ = 2.0°
Absorption correction: multi-scan (*SADABS*; Sheldrick, 1996)	*h* = −13→12
*T*_min_ = 0.943, *T*_max_ = 0.975	*k* = −13→13
11368 measured reflections	*l* = −11→21

### Refinement

**Table:**

Refinement on *F*^2^	Primary atom site location: structure-invariant direct methods
Least-squares matrix: full	Secondary atom site location: difference Fourier map
*R*[*F*^2^ > 2σ(*F*^2^)] = 0.067	Hydrogen site location: inferred from neighbouring sites
*wR*(*F*^2^) = 0.251	H-atom parameters constrained
*S* = 1.03	*w* = 1/[σ^2^(*F*_o_^2^)]
3913 reflections	(Δ/σ)_max_ < 0.001
307 parameters	Δρ_max_ = 0.25 e Å^−^^3^
0 restraints	Δρ_min_ = −0.28 e Å^−^^3^

### Special details

**Table:**

Geometry. All e.s.d.'s (except the e.s.d. in the dihedral angle between two l.s. planes) are estimated using the full covariance matrix. The cell e.s.d.'s are taken into account individually in the estimation of e.s.d.'s in distances, angles and torsion angles; correlations between e.s.d.'s in cell parameters are only used when they are defined by crystal symmetry. An approximate (isotropic) treatment of cell e.s.d.'s is used for estimating e.s.d.'s involving l.s. planes.
Refinement. Refinement of *F*^2^ against ALL reflections. The weighted *R*-factor *wR* and goodness of fit *S* are based on *F*^2^, conventional *R*-factors *R* are based on *F*, with *F* set to zero for negative *F*^2^. The threshold expression of *F*^2^ > σ(*F*^2^) is used only for calculating *R*-factors(gt) *etc*. and is not relevant to the choice of reflections for refinement. *R*-factors based on *F*^2^ are statistically about twice as large as those based on *F*, and *R*- factors based on ALL data will be even larger.

### Fractional atomic coordinates and isotropic or equivalent isotropic displacement parameters (Å^2^)

**Table:**

	*x*	*y*	*z*	*U*_iso_*/*U*_eq_	
Cl1	0.72987 (15)	0.38409 (13)	0.15930 (11)	0.0740 (6)	
O1	0.5201 (3)	1.0941 (3)	0.2030 (2)	0.0537 (10)	
O2	0.5296 (3)	1.0511 (3)	0.0810 (2)	0.0620 (11)	
O3	0.0934 (3)	0.7208 (3)	0.0853 (2)	0.0498 (10)	
O4	0.2460 (3)	0.6755 (3)	0.1814 (2)	0.0502 (10)	
N1	0.3811 (4)	0.8428 (4)	0.2747 (3)	0.0503 (12)	
H1	0.3691	0.7767	0.2525	0.060*	
N2	0.2308 (4)	0.9098 (4)	−0.0373 (3)	0.0517 (12)	
H2	0.3081	0.9226	−0.0367	0.062*	
C1	0.5027 (5)	1.0208 (4)	0.1418 (4)	0.0487 (14)	
C2	0.4502 (4)	0.9143 (4)	0.1609 (3)	0.0385 (12)	
C3	0.4320 (4)	0.9225 (4)	0.2347 (3)	0.0388 (12)	
C4	0.4770 (5)	1.0372 (4)	0.2651 (3)	0.0458 (13)	
H4A	0.4105	1.0809	0.2810	0.055*	
H4B	0.5438	1.0287	0.3085	0.055*	
C5	0.2134 (4)	0.7262 (4)	0.1207 (3)	0.0407 (13)	
C6	0.2842 (4)	0.7961 (4)	0.0774 (3)	0.0384 (12)	
C7	0.2060 (4)	0.8381 (4)	0.0164 (3)	0.0414 (13)	
C8	0.0777 (4)	0.7922 (5)	0.0181 (3)	0.0482 (14)	
H8A	0.0203	0.8548	0.0217	0.058*	
H8B	0.0473	0.7471	−0.0273	0.058*	
C9	0.4220 (4)	0.8188 (4)	0.1024 (3)	0.0403 (13)	
H9	0.4504	0.8481	0.0567	0.048*	
C10	0.4950 (4)	0.7072 (4)	0.1233 (3)	0.0379 (12)	
C11	0.4592 (5)	0.6052 (4)	0.0851 (3)	0.0508 (15)	
H11	0.3858	0.6039	0.0497	0.061*	
C12	0.5286 (5)	0.5052 (5)	0.0977 (3)	0.0560 (15)	
H12	0.5015	0.4371	0.0723	0.067*	
C13	0.6382 (5)	0.5084 (4)	0.1484 (3)	0.0474 (14)	
C14	0.6747 (5)	0.6063 (5)	0.1892 (3)	0.0526 (15)	
H14	0.7467	0.6066	0.2258	0.063*	
C15	0.6030 (4)	0.7050 (5)	0.1754 (3)	0.0469 (14)	
H15	0.6290	0.7720	0.2024	0.056*	
C16	0.3439 (5)	0.8482 (4)	0.3466 (3)	0.0442 (13)	
C17	0.2643 (5)	0.7626 (5)	0.3627 (3)	0.0541 (15)	
H17	0.2386	0.7049	0.3270	0.065*	
C18	0.2233 (6)	0.7624 (5)	0.4310 (4)	0.0669 (18)	
H18	0.1689	0.7049	0.4408	0.080*	
C19	0.2606 (5)	0.8448 (5)	0.4849 (4)	0.0625 (17)	
H19	0.2332	0.8430	0.5315	0.075*	
C20	0.3380 (5)	0.9292 (5)	0.4697 (3)	0.0584 (16)	
H20	0.3625	0.9862	0.5060	0.070*	
C21	0.3816 (5)	0.9328 (5)	0.4016 (3)	0.0526 (15)	
H21	0.4356	0.9911	0.3925	0.063*	
C22	0.1487 (5)	0.9681 (5)	−0.0949 (3)	0.0485 (14)	
C23	0.0411 (6)	0.9218 (5)	−0.1334 (4)	0.0694 (19)	
H23	0.0189	0.8469	−0.1224	0.083*	
C24	−0.0353 (6)	0.9854 (6)	−0.1886 (4)	0.0719 (19)	
H24	−0.1087	0.9525	−0.2137	0.086*	
C25	−0.0056 (6)	1.0951 (5)	−0.2070 (4)	0.0646 (17)	
H25	−0.0576	1.1376	−0.2439	0.078*	
C26	0.1045 (6)	1.1405 (5)	−0.1688 (4)	0.0619 (17)	
H26	0.1276	1.2147	−0.1806	0.074*	
C27	0.1799 (5)	1.0788 (5)	−0.1142 (3)	0.0543 (15)	
H27	0.2536	1.1116	−0.0895	0.065*	

### Atomic displacement parameters (Å^2^)

**Table:**

	*U*^11^	*U*^22^	*U*^33^	*U*^12^	*U*^13^	*U*^23^
Cl1	0.0781 (11)	0.0616 (10)	0.0802 (14)	0.0182 (8)	0.0080 (10)	0.0048 (9)
O1	0.063 (2)	0.047 (2)	0.054 (3)	−0.0074 (18)	0.020 (2)	−0.006 (2)
O2	0.068 (3)	0.067 (3)	0.057 (3)	−0.015 (2)	0.027 (2)	0.007 (2)
O3	0.039 (2)	0.059 (2)	0.049 (2)	−0.0080 (16)	0.0042 (19)	0.011 (2)
O4	0.055 (2)	0.059 (2)	0.037 (2)	−0.0088 (17)	0.0071 (19)	0.0074 (19)
N1	0.067 (3)	0.047 (3)	0.041 (3)	−0.008 (2)	0.020 (3)	−0.007 (2)
N2	0.039 (2)	0.063 (3)	0.051 (3)	−0.001 (2)	0.002 (2)	0.018 (3)
C1	0.043 (3)	0.047 (3)	0.059 (4)	−0.001 (2)	0.016 (3)	0.001 (3)
C2	0.026 (2)	0.056 (3)	0.033 (3)	−0.002 (2)	0.003 (2)	−0.003 (3)
C3	0.035 (3)	0.041 (3)	0.041 (3)	−0.001 (2)	0.007 (3)	0.001 (3)
C4	0.050 (3)	0.052 (3)	0.037 (3)	0.000 (3)	0.013 (3)	−0.002 (3)
C5	0.034 (3)	0.048 (3)	0.040 (3)	−0.004 (2)	0.005 (3)	−0.004 (3)
C6	0.033 (2)	0.046 (3)	0.036 (3)	−0.003 (2)	0.005 (2)	−0.003 (3)
C7	0.042 (3)	0.045 (3)	0.038 (3)	−0.004 (2)	0.008 (3)	−0.002 (3)
C8	0.040 (3)	0.058 (3)	0.045 (4)	−0.006 (2)	0.003 (3)	0.007 (3)
C9	0.033 (3)	0.051 (3)	0.037 (3)	−0.007 (2)	0.006 (2)	−0.001 (3)
C10	0.039 (3)	0.045 (3)	0.029 (3)	−0.002 (2)	0.005 (2)	−0.007 (3)
C11	0.041 (3)	0.065 (4)	0.041 (4)	−0.004 (3)	−0.009 (3)	−0.010 (3)
C12	0.054 (3)	0.058 (4)	0.054 (4)	0.001 (3)	0.003 (3)	−0.011 (3)
C13	0.048 (3)	0.045 (3)	0.049 (4)	0.002 (2)	0.008 (3)	0.000 (3)
C14	0.041 (3)	0.069 (4)	0.044 (4)	−0.002 (3)	−0.005 (3)	−0.001 (3)
C15	0.042 (3)	0.051 (3)	0.047 (4)	−0.004 (3)	0.004 (3)	−0.009 (3)
C16	0.051 (3)	0.050 (3)	0.032 (3)	0.005 (3)	0.009 (3)	0.003 (3)
C17	0.058 (3)	0.064 (4)	0.041 (4)	−0.015 (3)	0.011 (3)	−0.004 (3)
C18	0.070 (4)	0.077 (4)	0.058 (5)	−0.013 (3)	0.023 (4)	−0.002 (4)
C19	0.064 (4)	0.083 (4)	0.041 (4)	0.005 (3)	0.010 (3)	0.008 (4)
C20	0.071 (4)	0.064 (4)	0.039 (4)	0.007 (3)	0.005 (3)	−0.004 (3)
C21	0.061 (4)	0.056 (3)	0.040 (4)	−0.009 (3)	0.007 (3)	−0.005 (3)
C22	0.051 (3)	0.054 (3)	0.039 (3)	0.001 (3)	0.004 (3)	0.005 (3)
C23	0.065 (4)	0.063 (4)	0.068 (5)	−0.020 (3)	−0.020 (4)	0.014 (4)
C24	0.060 (4)	0.087 (5)	0.061 (5)	−0.016 (3)	−0.013 (4)	0.009 (4)
C25	0.060 (4)	0.072 (4)	0.058 (4)	0.014 (3)	0.002 (3)	0.010 (4)
C26	0.070 (4)	0.055 (4)	0.061 (4)	0.002 (3)	0.012 (4)	0.007 (3)
C27	0.051 (3)	0.060 (4)	0.051 (4)	0.000 (3)	0.004 (3)	−0.001 (3)

### Geometric parameters (Å, °)

**Table:**

Cl1—C13	1.745 (5)	C11—H11	0.9300
O1—C1	1.366 (6)	C12—C13	1.369 (7)
O1—C4	1.436 (6)	C12—H12	0.9300
O2—C1	1.223 (6)	C13—C14	1.367 (7)
O3—C5	1.357 (6)	C14—C15	1.383 (7)
O3—C8	1.439 (6)	C14—H14	0.9300
O4—C5	1.224 (6)	C15—H15	0.9300
N1—C3	1.345 (6)	C16—C17	1.384 (7)
N1—C16	1.412 (6)	C16—C21	1.394 (7)
N1—H1	0.8600	C17—C18	1.369 (8)
N2—C7	1.329 (6)	C17—H17	0.9300
N2—C22	1.410 (6)	C18—C19	1.362 (8)
N2—H2	0.8600	C18—H18	0.9300
C1—C2	1.427 (7)	C19—C20	1.353 (8)
C2—C3	1.366 (7)	C19—H19	0.9300
C2—C9	1.512 (7)	C20—C21	1.381 (8)
C3—C4	1.484 (7)	C20—H20	0.9300
C4—H4A	0.9700	C21—H21	0.9300
C4—H4B	0.9700	C22—C23	1.365 (7)
C5—C6	1.436 (7)	C22—C27	1.385 (7)
C6—C7	1.349 (7)	C23—C24	1.384 (8)
C6—C9	1.522 (6)	C23—H23	0.9300
C7—C8	1.509 (6)	C24—C25	1.364 (8)
C8—H8A	0.9700	C24—H24	0.9300
C8—H8B	0.9700	C25—C26	1.381 (8)
C9—C10	1.530 (6)	C25—H25	0.9300
C9—H9	0.9800	C26—C27	1.360 (8)
C10—C15	1.369 (7)	C26—H26	0.9300
C10—C11	1.383 (6)	C27—H27	0.9300
C11—C12	1.381 (7)		
			
C1—O1—C4	108.2 (4)	C10—C11—H11	118.8
C5—O3—C8	108.8 (4)	C13—C12—C11	118.6 (5)
C3—N1—C16	131.5 (5)	C13—C12—H12	120.7
C3—N1—H1	114.2	C11—C12—H12	120.7
C16—N1—H1	114.2	C14—C13—C12	120.9 (5)
C7—N2—C22	129.4 (4)	C14—C13—Cl1	121.0 (5)
C7—N2—H2	115.3	C12—C13—Cl1	118.2 (4)
C22—N2—H2	115.3	C13—C14—C15	119.0 (5)
O2—C1—O1	120.4 (5)	C13—C14—H14	120.5
O2—C1—C2	129.0 (5)	C15—C14—H14	120.5
O1—C1—C2	110.5 (5)	C10—C15—C14	122.2 (5)
C3—C2—C1	107.7 (5)	C10—C15—H15	118.9
C3—C2—C9	131.7 (5)	C14—C15—H15	118.9
C1—C2—C9	120.6 (5)	C17—C16—C21	118.5 (5)
N1—C3—C2	127.3 (5)	C17—C16—N1	116.8 (5)
N1—C3—C4	124.2 (5)	C21—C16—N1	124.7 (5)
C2—C3—C4	108.4 (4)	C18—C17—C16	120.3 (6)
O1—C4—C3	105.2 (4)	C18—C17—H17	119.8
O1—C4—H4A	110.7	C16—C17—H17	119.8
C3—C4—H4A	110.7	C19—C18—C17	121.3 (6)
O1—C4—H4B	110.7	C19—C18—H18	119.4
C3—C4—H4B	110.7	C17—C18—H18	119.4
H4A—C4—H4B	108.8	C20—C19—C18	118.9 (6)
O4—C5—O3	119.6 (4)	C20—C19—H19	120.5
O4—C5—C6	129.9 (5)	C18—C19—H19	120.5
O3—C5—C6	110.4 (5)	C19—C20—C21	121.8 (6)
C7—C6—C5	107.9 (4)	C19—C20—H20	119.1
C7—C6—C9	129.1 (5)	C21—C20—H20	119.1
C5—C6—C9	123.0 (5)	C20—C21—C16	119.2 (5)
N2—C7—C6	128.4 (5)	C20—C21—H21	120.4
N2—C7—C8	123.0 (5)	C16—C21—H21	120.4
C6—C7—C8	108.6 (5)	C23—C22—C27	117.9 (5)
O3—C8—C7	104.2 (4)	C23—C22—N2	124.2 (5)
O3—C8—H8A	110.9	C27—C22—N2	118.0 (5)
C7—C8—H8A	110.9	C22—C23—C24	120.6 (6)
O3—C8—H8B	110.9	C22—C23—H23	119.7
C7—C8—H8B	110.9	C24—C23—H23	119.7
H8A—C8—H8B	108.9	C25—C24—C23	121.6 (6)
C2—C9—C6	113.4 (4)	C25—C24—H24	119.2
C2—C9—C10	114.5 (4)	C23—C24—H24	119.2
C6—C9—C10	112.1 (4)	C24—C25—C26	117.4 (6)
C2—C9—H9	105.2	C24—C25—H25	121.3
C6—C9—H9	105.2	C26—C25—H25	121.3
C10—C9—H9	105.2	C27—C26—C25	121.3 (6)
C15—C10—C11	116.9 (5)	C27—C26—H26	119.3
C15—C10—C9	122.2 (4)	C25—C26—H26	119.3
C11—C10—C9	120.7 (4)	C26—C27—C22	121.1 (6)
C12—C11—C10	122.3 (5)	C26—C27—H27	119.4
C12—C11—H11	118.8	C22—C27—H27	119.4
			
C4—O1—C1—O2	178.2 (5)	C7—C6—C9—C10	−133.2 (5)
C4—O1—C1—C2	−0.8 (5)	C5—C6—C9—C10	49.9 (6)
O2—C1—C2—C3	−177.5 (5)	C2—C9—C10—C15	−20.6 (7)
O1—C1—C2—C3	1.3 (6)	C6—C9—C10—C15	−151.7 (5)
O2—C1—C2—C9	1.1 (8)	C2—C9—C10—C11	164.8 (4)
O1—C1—C2—C9	180.0 (4)	C6—C9—C10—C11	33.7 (7)
C16—N1—C3—C2	−170.5 (5)	C15—C10—C11—C12	−0.6 (8)
C16—N1—C3—C4	8.6 (8)	C9—C10—C11—C12	174.3 (5)
C1—C2—C3—N1	177.9 (5)	C10—C11—C12—C13	−1.7 (8)
C9—C2—C3—N1	−0.5 (9)	C11—C12—C13—C14	4.0 (8)
C1—C2—C3—C4	−1.3 (5)	C11—C12—C13—Cl1	−176.0 (4)
C9—C2—C3—C4	−179.7 (5)	C12—C13—C14—C15	−3.9 (8)
C1—O1—C4—C3	0.0 (5)	Cl1—C13—C14—C15	176.1 (4)
N1—C3—C4—O1	−178.4 (4)	C11—C10—C15—C14	0.8 (8)
C2—C3—C4—O1	0.9 (5)	C9—C10—C15—C14	−174.1 (5)
C8—O3—C5—O4	176.8 (5)	C13—C14—C15—C10	1.5 (8)
C8—O3—C5—C6	−2.7 (5)	C3—N1—C16—C17	162.7 (5)
O4—C5—C6—C7	−176.9 (5)	C3—N1—C16—C21	−17.4 (9)
O3—C5—C6—C7	2.5 (6)	C21—C16—C17—C18	0.6 (8)
O4—C5—C6—C9	0.5 (8)	N1—C16—C17—C18	−179.5 (5)
O3—C5—C6—C9	179.9 (4)	C16—C17—C18—C19	−0.9 (9)
C22—N2—C7—C6	−169.7 (5)	C17—C18—C19—C20	1.1 (9)
C22—N2—C7—C8	8.8 (9)	C18—C19—C20—C21	−1.1 (9)
C5—C6—C7—N2	177.4 (5)	C19—C20—C21—C16	0.8 (9)
C9—C6—C7—N2	0.2 (9)	C17—C16—C21—C20	−0.6 (8)
C5—C6—C7—C8	−1.2 (6)	N1—C16—C21—C20	179.6 (5)
C9—C6—C7—C8	−178.4 (5)	C7—N2—C22—C23	−37.0 (9)
C5—O3—C8—C7	1.9 (5)	C7—N2—C22—C27	143.6 (6)
N2—C7—C8—O3	−179.0 (5)	C27—C22—C23—C24	−1.5 (9)
C6—C7—C8—O3	−0.3 (6)	N2—C22—C23—C24	179.1 (6)
C3—C2—C9—C6	65.9 (7)	C22—C23—C24—C25	0.7 (10)
C1—C2—C9—C6	−112.3 (5)	C23—C24—C25—C26	0.5 (10)
C3—C2—C9—C10	−64.5 (7)	C24—C25—C26—C27	−0.8 (9)
C1—C2—C9—C10	117.3 (5)	C25—C26—C27—C22	−0.1 (9)
C7—C6—C9—C2	95.1 (7)	C23—C22—C27—C26	1.2 (9)
C5—C6—C9—C2	−81.7 (6)	N2—C22—C27—C26	−179.4 (5)

### Hydrogen-bond geometry (Å, °)

**Table:**

*D*—H···*A*	*D*—H	H···*A*	*D*···*A*	*D*—H···*A*
N1—H1···O4	0.86	2.05	2.797 (3)	145
N2—H2···O2^i^	0.86	2.09	2.907 (3)	158
C4—H4A···O4^ii^	0.97	2.24	3.206 (3)	178
C8—H8A···Cg5^iii^	0.97	2.98	3.721 (3)	134
C8—H8B···Cg4^iv^	0.97	2.91	3.617 (3)	131
C19—H19···Cg3^v^	0.93	2.90	3.766 (3)	156

Symmetry codes: (i) −*x*+1, −*y*+2, −*z*; (ii) −*x*+1/2, *y*+1/2, −*z*+1/2; (iii) −*x*+1, −*y*+1, −*z*+1; (iv) *x*−1/2, −*y*−1/2, *z*−1/2; (v) *x*−1/2, −*y*−1/2, *z*−3/2.
